# The effects of immediate and delayed feedback on hand hygiene compliance

**DOI:** 10.1186/2047-2994-4-S1-P287

**Published:** 2015-06-16

**Authors:** E Gavrieli, A Drach-Zahavy, I Tal, G Rahav, D Ben-David

**Affiliations:** 1 Infectious Diseases Depatment, Sheba Medical Center, Ramat Gan, Israel; 2 Faculty of social welfare and health science, Haifa University, Haifa, Israel

## Introduction

Audit and feedback is widely used as a part of a multimodal strategy to improve hand hygiene (HH) compliance. Few studies have investigated the effect of different feedback strategies.

## Objectives

To compare delayed versus immediate feedback.

## Methods

A prospective 5-step interventional study was conducted between 2012-2014 in 2 pediatric intensive care units (PICU and HPICU) at a tertiary medical center in Israel. The intervention steps included (1) baseline observations (2) training (3) providing delayed feedback in PICU versus both delayed and immediate feedback in HPICU (4) providing immediate feedback in PICU (5) final assessment stage. HH observations were conducted according to the 5-moment HH model. A Mixed Linear regression analysis was used to examine the models with repeated measurements. Each stage was defined relative to the baseline stage in 5 moments. In addition, each stage was defined in comparison to the previous stage

## Results

A total of 8,159 observations were completed during the study period. HPICU HH compliance increased from 33.4% in the baseline stage to 71.8% at the final stage; PICU HH compliance increased from 30.92% to 67.1%. When each stage was compared to the baseline, HH compliance rates of all WHO’s 5-moment were significantly improved (p<.0001). Assessment the impact of each step showed that only immediate feedback was associated with a significant increase in HH compliance before clean/aseptic contact (HPICU 16.3% (step 2) versus 45.0% (step 3); PICU 25.8 %(step 3) versus 48.4% (step 4), p<0.001)).

## Conclusion

Implementation of a multifaceted intervention was associated with sustained improvement in HH adherence. Delayed feedback contributed to a slow but gradual increase at all stages, while immediate feedback contributed to a significant increase in HH compliance before clean/aseptic contact

## Disclosure of interest

None declared.

